# Risk Communication Through Health Beliefs for Preventing Opisthorchiasis-Linked Cholangiocarcinoma: A Community-Based Intervention in Multicultural Areas of Thailand

**DOI:** 10.31557/APJCP.2021.22.10.3181

**Published:** 2021-10

**Authors:** Nopparat Songserm, Pariwat Korsura, Somkiattiyos Woradet, Akhtar Ali

**Affiliations:** 1 *Department of Community Health, Faculty of Public Health, Ubon Ratchathani Rajabhat University, Ubon Ratchathani, Thailand. *; 2 *Somboon Sub-district Health Promoting Hospital, Khukhan District, Sisaket, Thailand. *; 3 *Department of Public Health, Faculty of Health and Sports Science, Thaksin University, Phatthalung Province, Thailand. *; 4 *Department of Biological Science, The University of Tulsa, Oklahoma, United State of America. *

**Keywords:** Health education, risk communication, health beliefs, opisthorchiasis, cholangiocarcinoma

## Abstract

**Objective:**

This research aimed to study the effects of the risk communication program through the Cambodian folk song to prevent Opisthorchiasis-linked cholangiocarcinoma (OV-CCA).

**Methods:**

We conducted the quasi-experimental research between August and December 2017 in the Cambodian communities, one-fourth of ethnic minorities residing in multicultural areas of Sisaket Province, Thailand. The samples consisted of 94 equally people divided into experimental group and control group. The experimental group included 47 people at-risk of OV-CCA who received the program for 12 weeks, while the control group received regular services. We collected data by using a questionnaire with a reliability of 0.93. Descriptive and inferential statistics were used for data analysis.

**Results:**

The study indicated that the socioeconomic information of both groups was not different. The mean scores of all issues (health beliefs, social support, and prevention behavior in the experimental group were higher than those of the control group with statistical significance. Closer inspection showed that the mean difference of the health beliefs was 55.61 points (95%CI: 52.39-57.42, p<0.001), social support was 9.09 points (95%CI: 8.12-10.05, p<0.001), and prevention behavior was 6.38 points (95%CI: 5.43-7.33, p<0.001).

**Conclusion:**

Through the Cambodian folk song, the risk communication program by applying the health beliefs and social support to prevent OV-CCA is beneficial for behavior modification in areas with similar cultures.

## Introduction

Opisthorchiasis-linked cholangiocarcinoma (OV-CCA) occurs from eating raw or undercooked fish. It has been classified as a group 1 carcinogen in humans since 1994 (IARC, 1994). At present, although it is still a significant public health problem in some parts of the world, it is neglected until it becomes a parasitic disease (Sripa, 2008). Liver fluke (O. viverrini, OV) enters the human body by eating freshwater cyprinid fish with metacercaria from undercooked food (Sripa et al., 2018; Songserm et al., 2012). About 6 million Thai people out of 10 million people worldwide are at risk of OV infection, most of whom live in the northeast region (IARC, 2012).

A 2009 survey of OV prevalence in northeast Thailand found that the Sisaket Province was the top three provinces with the highest prevalence (Sithithaworn et al., 2012). Similarly, a spatial epidemiological survey between 2013-2016 in the Sisaket found a prevalence of 38.6%, 19.2%, 12.9%, and 13.1%, respectively (Sisaket Provincial Public Health Office, 2016). Moreover, 12.7% of the OV prevalence was reported in the Khukhan District, a multicultural area of four tribes (Suay, Cambodian, Laos, and Yerr). More importantly, the highest prevalence of OV was found in the Cambodian community (16.7%) (Khukhan District Public Health Office, 2016). It indicated that most OV prevalence in all areas was higher than the 5% standard.

The Ministry of Public Health is the leading organization in implementing the strategy called “Get rid of OV and reduce CCA in Isan people (north-eastern people)” through five main measures (Jongsuksuntigul and Imsomboon, 2003; Sripa et al., 2011). The policies, measurements, and operational goals are defined as follows. Regarding disease prevention and screening, the risk behavior and OV prevalence must not be more than 5%. However, the strategy set was not practical. It may be caused by various beliefs and behaviors of people in the area that have been around and rooted for a long time. Therefore, it is needed to find ways to correctly change the people who are not against the culture and views that have been in the community for a long time. More importantly, the methods should maintain the roles and responsibilities of the community in all six areas: (1) being a spiritual anchor, (2) determining and controlling society, (3) being a tool for the integration of children and grandchildren, and the unity of the people in the village, (4) being a place for education, (5) being a means of entertainment for people in the community, and (6) creating an identity for locality (Khowdee, 2012).

One of the rituals of the Cambodian people living in the Sisaket is called Jolmamuad (a Cambodian folksong). It resembles a medium and uses musical instruments to perform rituals, aiming to treat sick people who have been treated by usual methods or at hospitals or by modern medicine but have not recovered (Bertrand, 1997). It is considering alternative medicine that should help heal (especially psychological aspects) because patients undergo treatment by other methods that have not been worked out (Eisenbruch, 2019). Risk communication through health beliefs is beneficial for alternative medicine and disease prevention from public health. Therefore, this study aimed to compare the effects of the risk communication program through the Cambodian folksong by applying the health belief model of Becker (1974) and social support to prevent OV-CCA in Cambodian communities in the Sisaket before and after the experiment within and between the experimental group and the control group. 

## Materials and Methods

*Study design*

The quasi-experimental research (two-group pre-posttest design) was carried out. The study period was between August and December 2017. The samples were divided into two groups. The experimental group received the risk communication program through the Cambodian folksong by applying the health beliefs and social support. In contrast, the control group received regular services from Sub-District Health Promoting Hospital. Data were collected before and after the experiment for 12 weeks. 

*Study participants*

**Figure 1:**
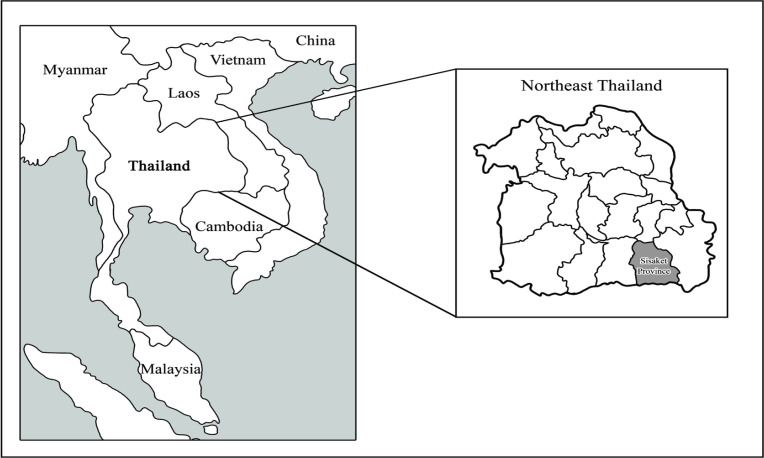
The Research Area of the Study. The Sisaket Province is a multicultural area of Thailand's four tribes (Suay, Cambodian, Laos, and Yerr)

This study’s population was Thai-Cambodian people aged 20 years and over living in the Khukhan District, Sisaket Province (Figure 1). The study participants were briefed about the research’s purpose, and their informed consent was obtained before data collection. They were screened from August - December 2017 and found to have at-risk eating habits and liver fluke eggs detected in their stool. 

**Figure 2:**
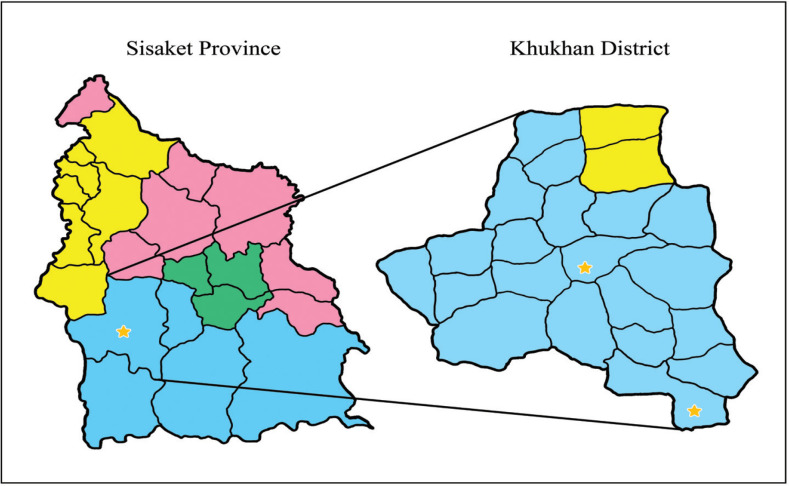
Determination of the Study Setting. The yellow areas represent the Suay Communities; the blue areas represent the Cambodian Communities; the pink areas represent the Laos Communities; the green places represent the Yerr Communities. The star symbols represent the areas randomly selected for this study

We employed the formula for calculating sample size to compare mean values for two independent population groups. This study’s sample size was 47 people per group, randomly selected from a three-stage sampling method. Step 1: The samples’ characteristics were defined by specifying 27 Sub-district Health Promoting Hospitals in Khukhan District, Sisaket Province. Step 2: the samples were randomly selected from Sub-District Health Promoting Hospitals by drawing lots two times. The first time was for designating the experimental group, which the Somboon Sub-District Health Promoting Hospital was selected. The second time was setting the control group, which the Prue Khan Sub-District Health Promoting Hospital was established. Step 3: A simple random sampling method was employed to select 47 samples based on the inclusion criteria from both areas equally (Figure 2). 

Inclusion criteria

1. Those who were at-risk eating habits for OV infection had been detected with OV eggs in feces.

2. Male and female aged 20 years and over.

3. The persons who must be able to read and write without the problems of hearing, speaking, and vision.

4. The ones were giving consent and cooperation in participation in the study. 

Exclusion criteria

1. Those who cannot join every activity.

*Variables, tools, and technique data collection*

The research tools consisted of 2 parts: the experiment’s tools and the tools used for data collection. 

1. The tools used in the experiment: It was the risk communication program through the Cambodian folksong by applying the health beliefs and social support to prevent OV-CCA. The content consisted of OV-CCA knowledge and the pictures of the Cambodian folk song, slide show, slide for the lecture, video, and computer. The activities were as follows. 

• Week 1: “Preparing the leaders to create a village model”: The activities included watching a video together with the Cambodian folksong, a lecture by the speaker about the knowledge and practice for preventing OV-CCA, handing out manuals, and sharing of knowledge among participants.

• Week 2: “The leaders can do”: It consisted of activities of watching a video about OV-CCA together with the Cambodian folksong, a lecture by the speaker about the risk and the severity of OV-CCA, a presentation to gain awareness of the seriousness of OV-CCA, and knowledge exchange about the practice and group discussion. 

• Week 3: “Disseminating knowledge to the community”: The activities included a lecture by the speaker about the benefits and barriers in practice together with the Cambodian folksong and exchange of knowledge and problems in the method of preventing OV-CCA. 

• Week 4-12: “Home visits”: Social support was given by the researcher, staff of Sub-District Health Promoting Hospital, and village health volunteers. We did home visits to provide information, knowledge, and encouragement to family members and the group members at risk of having OV-CCA. It was a way to encourage patients to care about OV-CCA prevention by supporting patients with correct practices and compliments. At-risk groups were encouraged to have health screenings every year. We operated home visits on weeks 5, 7, 9, and 11. The members were asked to answer a questionnaire in week 12. The stool examination for OV was also tested in week 12.

2. The tools used for data collection: It was a questionnaire that the researcher and the research assistant used to ask the experimental group and the control group. It was divided into four parts: Part 1 sociodemographic information questionnaire, Part 2 health belief questionnaire, Part 3 social support questionnaire, and Part 4 prevention behavior of OV-CCA questionnaire. The tools were developed and examined for quality as follows. (1) Data were studied and collected from documents, journals, concepts, theories, and related research based on the research framework to be the information for constructing a questionnaire. (2) The content validity was checked by five experts (two public health personnel who work as public health officials and three who work in the Sub-District Health Promoting Hospitals). Index of Item-Objective Congruence (IOC) was investigated for the consistency between the purpose and the content. The IOC values of 0.50 and above are considered to be consistent. (3) The reliability was verified by trying out the questionnaire with 30 people at-risk of OV-CCA. Their qualifications were similar to the samples in the Kwang Khao Sub-District, Khukhan District, Sisaket Province. The results were analyzed to improve the quality of the questionnaire according to the criteria. For the knowledge category, the reliability was determined by calculating the K-R 20 value of Kuder-Richardson, and the value was 0.91. The reliability of the health belief consisted of 4 parts, including (1) perceived risk of OV-CCA, which was 0.89, (2) perceived severity of OV-CCA, which was 0.90, (3) perceived benefits of OV-CCA prevention which was 0.85, and (4) perceived barriers of OV-CCA prevention which was 0.92. The reliability of the practice for OV-CCA prevention was 0.94. The reliability of the whole questionnaire was 0.93.

*Statistical analysis*

A statistical software package was employed for data analysis, SPSS version 26.0 (IBM Company, Chicago, USA). The reliability of the statistical testing was set at 0.05. Descriptive statistics, including number, percentage, mean, standard deviation, minimum, and maximum values, were used to analyze the samples’ sociodemographic characteristics. In addition, the following inferential statistics were employed. A comparison of mean scores of the health belief model, social support, and OV-CCA prevention behavior within the experimental and control groups before and after the experiment was used with a paired t-test, while an independent t-test was used to test between both groups.

## Results

Table 1 presents sociodemographic characteristics and the history of risk behavior for OV-CCA between the experimental group and the control group. The basic information, namely the proportion of male and female, age, marital status, educational level, and family income of most samples in both groups, was not different. We obtained the history of risk behavior for OV-CCA from 3 assessments. (1) 14.89% of the experimental group and 25.53% of the control group had a history of eating food prepared from raw or undercooked freshwater cyprinid fish, most of which were from freshwater sources. (2) 14.89% of the experimental group and 25.53% of the control group used to buy praziquantel for themselves. Also, (3) 28.57% of the experimental group and 58.33% of the control group often drank alcohol while eating raw fish. 

The comparison of the mean scores of the health belief model for promoting OV-CCA prevention behavior is presented in Table 2. It was found that the overall mean score of the health belief model of the experimental group on OV-CCA prevention was higher than that of the control group with statistical significance. The mean difference was 55.61 points (95%CI: 53.10-58.12). In addition, when separated in each issue, it was found that the mean scores for all matters (perceived risk, perceived severity, perceived benefits, and perceived barriers) of the experimental group after the program were higher than those of the control group with statistical significance (p<0.001).

The comparison of social support for OV-CCA prevention was also assessed in both groups (Table 3). After the experiment, the results showed that the mean score of social support for OV-CCA prevention was higher than that of the control group with statistical significance (p<0.001). The mean difference was 9.09 points (95% CI: 8.12-10.05).

Table 4 presents a comparison of the mean score of OV-CCA prevention behavior. It was found that after the experiment, the mean score of OV-CCA prevention behavior of the experimental group was higher than that of the control group with statistical significance (p<0.001). The mean difference was 6.38 points (95% CI: 5.43-7.33).

Table 5 compares the proportion of liver fluke infection before and after the experiment between the experimental and control groups. After the investigation, there was a statistically significant difference in the proportion of liver fluke infection between the experimental and control groups. The proportion of the experiment’s liver fluke infection was less than that of the comparison group by 19.15% (95% CI: 6.74-31.56, p=0.004).

**Table 1:** Sociodemographic Characteristics and History of Risk Behavior for Opisthorchiasis and Cholangiocarcinoma Between the Experimental Group and the Control Group

General information	Experimental group (n=47)		Control group (n=47)	
	Number	%	Number	%
Sex				
Male	27	57.45	31	65.96
Female	20	42.55	16	34.04
Age				
20 – 29 years	4	8.51	6	12.77
30 – 39 years	8	17.02	8	17.02
40 – 49 years	23	48.94	15	31.91
50 years and over	12	25.53	18	38.3
Mean (S.D.), Median (Min : Max)	44.13 (8.92), 44 (27 : 62)		44.19 (10.75), 44 (22 : 61)	
Marital status				
Single	6	12.77	12	25.53
Married	40	85.11	34	72.34
Widowed	1	2.12	1	2.13
Highest educational level				
Primary school	7	14.89	3	6.38
Junior high school	19	40.43	17	36.17
High school/vocational certificate	17	36.17	15	31.91
Diploma	2	4.26	11	23.4
Bachelor's degree or higher	2	4.26	1	2.13
Occupation				
Farmer	38	80.85	33	70.21
Seller	8	17.02	13	27.66
Employee	1	2.13	1	2.13
Average family income per month				
<5,000 baht	16	34.04	21	44.68
5,000 – 9,999 baht	24	51.06	17	36.17
10,000 baht and over	7	14.9	9	19.15
Mean (S.D.)	7,146.81 (4,677.46)		8,219.15 (10664.13)	
Having history of eating raw fish				
No	40	85.11	35	74.47
Yes	7	14.89	12	25.53
Bringing fish from fresh water sources	5	71.42	11	91.67
Eating with friends	6	85.71	10	83.33
Used to take praziquantel				
No	29	61.7	20	42.55
Yes	18	38.3	27	57.45
Drinking alcohol while eating raw fish				
No	5	71.43	5	41.67
Yes	2	28.57	7	58.33

**Table 2:** Comparison of the Differences of the Mean Scores of Health Beliefs in each and Overall Aspect before and after the Experiment between the Experimental Group and the Control Group

Perceived	Assessment period	Experimental group		Control group		Mean difference	95% CI	p-value
		Mean	S.D.	Mean	S.D.			
Risk	Before	51.77	3.29	51.34	3.52	0.43	-0.96, 1.82	0.546
	After	61.77	1.72	51.09	2.47	10.68	9.81, 11.55	<0.001
Severity	Before	49.61	3.44	49.7	0.42	-0.85	-1.38, 1.21	0.89
	After	60.08	2.49	49.38	2.38	10.7	9.70, 11.70	<0.001
Benefits	Before	47.82	3.37	47.95	3.79	-0.12	-1.59, 1.34	0.864
	After	66.17	2.82	46.82	3.65	19.34	18.00, 20.67	<0.001
Obstacles	Before	42.8	3.29	43.04	3.17	-0.23	-1.55, 1.09	0.727
	After	57.59	2.76	43.4	3.79	14.19	12.83, 15.55	<0.001
Overall	Before	192.02	7.1	192.04	7.04	-0.02	-2.91, 2.87	0.988
	After	245.61	5.54	190.7	6.67	55.61	52.39, 57.42	<0.001

**Table 3:** Comparison of the Mean Score of Social Support for the Prevention of Opisthorchiasis and Cholangiocarcinoma before and after the Experiment between the Experimental Group and the Control Group

Assessment period	Experimental group		Control group		Mean difference	95% CI	p-value
	Mean	S.D.	Mean	S.D.			
Before the experiment	16.79	3.58	18.43	3.04	-1.64	-2.99, -2.77	0.019
After the experiment	28.62	1.92	19.53	2.74	9.09	8.12, 10.05	<0.001

**Table 4:** Comparison of the Mean Score of Opisthorchiasis and Cholangiocarcinoma Prevention Behavior before and after the Experiment between the Experimental Group and the Control Group

Assessment period	Experimental group		Control group		Mean difference	95% CI	p-value
	Mean	S.D.	Mean	S.D.			
Before the experiment	23.21	2.44	22.65	2.51	0.55	-0.46, -1.56	0.282
After the experiment	29.68	2.34	23.29	2.30	6.38	5.43, 7.33	<0.001

**Table 5:** Comparison of the Proportion of Liver Fluke Infection before and after the Experiment between the Experimental Group and the Control Group

Assessment period	Experimental group		Control group		Proportion difference	95% CI	p-value
	No.	%	No.	%			
Before the experiment	7	14.89	9	19.15	-4.26	-19.43, -10.92	0.583
After the experiment	1	2.13	10	21.21	-19.15	-31.56, -6.74	0.004

Note, Analyzed by using proportion difference test

## Discussion

Based on the study of the effects of the risk communication program through the Cambodian folksong by applying the health beliefs and social support to prevent OV-CCA among Thai-Cambodian people residing in Sisaket Province, a multicultural area of four tribes (Suay, Cambodian, Laos, and Yerr) in Thailand. The Cambodian folksong is a ritual related to the treatment of patients of Cambodian people. It is a tradition that has been passed down for many generations (Khowdee, 2012). Ancestors of Cambodian people have used this wisdom to treat illnesses for themselves and those who live together in the community (Bertrand, 1997). Therefore, it is an essential and sacred ritual in the Cambodian community in Thailand. Based on the study, the mean score of those who perceived the health beliefs for preventing the OV-CCA after the experimental group’s experiment was higher than that of the control group with significant significance (p<0.001). It is consistent with the concept of health beliefs regarding the perceived risk of disease with the behavior that follows the healthcare staff’s advice and the practice of disease prevention of patients. This causes awareness of the severity of the illness (Deeprom and Sirisuwan, 2018). It is also consistent with comparing preventive behavior between the application of the health belief model with participation and a handbook for OV-CCA prevention among primary school students in the Roi-Et Province of Thailand. It was found that after the experiment, the score of perceived benefits of OV-CCA prevention of the experimental group was at a high level (Thongnamuang and Duangsong, 2012). According to the concept of health beliefs, people who find ways to prevent or treat themselves from the disease must believe that it is good and can cure the disease. Therefore, these people decide to follow the instructions, and they usually choose to do things that are considered to give better results than disadvantages and the trust in staff (Eisenbruch and Handelman, 1990).

The mean social support score for OV-CCA prevention behavior after the experimental group experiment was higher than that of the control group with statistical significance (p<0.001). It indicates that the experimental group had learned and changed behavior, resulting in awareness leading to practice that can be used in daily lives. Besides, the perceived benefits from activities in the prevention of OV-CCA can be transferred to the community. Social support is an influential factor and plays an essential role in people’s physical and mental health behavior (Thongnamuang and Duangsong, 2012). If these two concepts are applied together with OV-CCA prevention, people will change their behavior to prevent OV-CCA. It is consistent with the study of Kongsila et al. (2016), who developed a program for OV prevention in the community’s risk group using participatory techniques to organize activities. With this, the community was more interested and engaged with the activities. 

We also studied OV-CCA prevention behavior. After the experiment, the results showed that the experimental group’s mean score was higher than that of the control group with statistical significance (p<0.001). Thus, it shows that the risk communication program leads to better behavior modification for the OV-CCA prevention, which is consistent with the development of a program to prevent OV in the community by educating, publicizing, and campaigning leading to the practice of OV prevention (Kongsila et al., 2016; Deeprom and Sirisuwan, 2018).

In conclusion, through the Cambodian folk song, the risk communication program by applying the health beliefs and social support to prevent OV-CCA is beneficial for behavior modification in areas with similar cultures. It is also used to avoid diseases caused by other behaviors in at-risk people for enhancing the potential to take care of their health. Therefore, public health officials should adopt this program to prevent OV-CCA or other diseases among communities in other areas.

## Author Contribution Statement

NS and PK conceived and designed the research. NS and PK were responsible for connecting and coordinating the fieldwork. PK collected the data. NS and SW carried out the analyses. NS and AA reviewed drafts of the paper. All authors contributed to the writing and revisions of the manuscript and approved the final version.

## References

[B1] Becker MH (1974). The health belief model and sick role behavior. Health Educ Behav.

[B2] Bertrand D (1997). Mental health and cultural issues: the return of Khmers from France to Cambodia for healing purposes. Sante.

[B3] Deeprom C, Sirisuwan P (2018). Prevention behaviors of the liver fluke among screened people for liver fluke in Bansonghong, Rongkham Sub-District, Rongkham District, Kalasin Province. KKU J Public Health Res.

[B4] Eisenbruch M (2019). Putting the spirit into culturally responsive public health: explaining mass fainting in Cambodia. J Relig Health.

[B5] Eisenbruch M, Handelman L (1990). Cultural consultation for cancer: astrocytoma in a Cambodian adolescent. Soc Sci Med.

[B6] IARC (1994). Schistosomes, liver flukes and Helicobacter pylori IARC Working Group on the Evaluation of Carcinogenic Risks to Humans. Lyon. IARC Monogr Eval Carcinog Risks Hum.

[B7] IARC (2012). Opisthorchis viverrini and Clonorchis sinensis. IARC Working Group on the Evaluation of Carcinogenic Risks to Humans. Lyon. IARC Monogr Eval Carcinog Risks Hum.

[B8] Jongsuksuntigul P, Imsomboon T (2003). Opisthorchiasis control in Thailand. Acta Trop.

[B9] Khowdee S (2012). The role of Jolmamuad Rite in Khmer community, Surin Province. J Liberal Arts Ubon Ratchathani University.

[B11] Kongsila P, Saensak S, Thanasai J (2016). Effects of a program to prevent opisthorchiasis on knowledge and perceptions of health in the risk group. J Health Sci Res.

[B13] Sithithaworn P, Andrews RH, Nguyen VD (2012). The current status of opisthorchiasis and clonorchiasis in the Mekong Basin. Parasitol Int.

[B14] Songserm N, Promthet S, Sithithaworn P (2012). Risk factors for cholangiocarcinoma in high-risk area of Thailand: Role of lifestyle, diet and methylenetetrahydrofolate reductase polymorphisms. Cancer Epidemiol.

[B15] Sripa B (2008). Concerted action is needed to tackle liver fluke infections in Asia. PLoS Negl Trop Dis.

[B16] Sripa B, Bethony JM, Sithithaworn P (2011). Opisthorchiasis and Opisthorchis-associated cholangiocarcinoma in Thailand and Laos. Acta Trop.

[B17] Sripa B, Tangkawattana S, Brindley PJ (2018). Update on pathogenesis of opisthorchiasis and cholangiocarcinoma. Adv Parasitol.

[B18] Thongnamuang S, Duangsong R (2012). The effectiveness of application by health belief model and social support for preventive behavior of opisthorchiasis and cholangiocarcinoma among primary school students in Moeiwadi District, Roi-Et Province. KKU Res J (GS).

